# Early brain microstructural development among preterm infants requiring caesarean section versus those delivered vaginally

**DOI:** 10.21203/rs.3.rs-3389209/v1

**Published:** 2023-10-06

**Authors:** Pratheek S. Bobba, Clara F. Weber, Ajay Malhotra, Mert O. Bahtiyar, Joshua Copel, Sarah N. Taylor, Laura R. Ment, Seyedmehdi Payabvash

**Affiliations:** Yale School of Medicine; Lübeck University; Yale School of Medicine; Yale School of Medicine; Yale School of Medicine; Yale School of Medicine; Yale School of Medicine; Yale School of Medicine

## Abstract

It is known that the rate of caesarean section (C-section) has been increasing among preterm births. However, the relationship between C-section and long-term neurological outcomes is unclear. In this study, we utilized diffusion tensor imaging (DTI) to characterize the association of delivery method with brain white matter (WM) microstructural integrity in preterm infants. We retrospectively analyzed the DTI scans and health records of preterm infants without neuroimaging abnormality on pre-discharge term-equivalent MRI. We applied both voxel-wise and tract-based analyses to evaluate the association between delivery method and DTI metrics across WM tracts while controlling for numerous covariates. We included 68 preterm infants in this study (23 delivered vaginally, 45 delivered via C-section). Voxel-wise and tract-based analyses revealed significantly lower fractional anisotropy values and significantly higher diffusivity values across major WM tracts in preterm infants delivered via C-section when compared to those delivered vaginally. These results may be partially, but not entirely, mediated by lower birth weight among infants delivered by C-section. Nevertheless, these infants may be at risk for delayed neurodevelopment and could benefit from close neurological follow up for early intervention and mitigation of adverse long-term outcomes.

## INTRODUCTION

Preterm birth (< 37 weeks gestation) accounts for more than 10% of live births worldwide.(1) Prematurity is also the leading cause of infant mortality, contributing to approximately one million infant deaths each year.(2, 3) These infants suffer from a range of pathologies in the neonatal period including respiratory distress, intraventricular hemorrhage, and necrotizing enterocolitis among others.(4) Numerous studies have also linked prematurity to adverse long-term neurodevelopmental outcomes, including increased rates of cerebral palsy, cognitive impairment, and behavioral disorders in preterm infants when compared to term births.(5-7) The degree of prematurity has also been associated with increased risk of such adverse neurological outcomes.(8, 9) Notably, while C-sections account for more than a fifth of all live births globally, the rate of C-section in preterm births has risen from around 25% to over 35% in the past three decades.(10, 11) Thus given the increasing prevalence of C-section delivery, particularly in prematurity, a particular area of recent interest has been the relationship between delivery method and infant outcomes.

Multiple studies have found significant associations of C-section delivery with adverse cognitive outcomes, emotional problems, sleep problems, and increased risk of developing autism spectrum disorder and attention deficit hyperactivity disorder later in life.(12-15) Some of these adverse outcomes may – at least partially – be mediated by confounding maternal and infant factors such as birthweight, maternal age, maternal substance use and infant sex.(16, 17) In contrast, some studies report no association between delivery method and infant mortality, neurodevelopmental delay, or rate of neurological disorder development among term, extremely preterm or very low birth weight infants.(18-22) Given these conflicting reports, we aimed to examine the association of delivery method with preterm infants’ brain white matter (WM) microstructure, a biomarker of developmental disorders.

Although conventional brain MRIs can identify drastic structural and pathologic abnormalities, advanced MR techniques such as diffusion tensor imaging (DTI) can further characterize brain development by evaluating WM microstructural integrity. Prior studies have utilized DTI to characterize WM microstructure in infants across a range of gestational ages, identifying specific WM regions that are microstructurally delayed in infants of lower gestational age.(23, 24) We have recently shown the associations of gestational age at birth and corrected age at the time of scan on infants’ brain WM microstructural development.(23) Deoni et al. have also utilized DTI to study how delivery method is associated with infant brain development in full-term, healthy infants, finding that those born via C-section displayed signs of lower WM development at two weeks. However, they noted that such differences seemed to be less apparent later in life.(25)

In this study, we compared the early WM microstructural integrity of radiologically normal singleton preterm infants born via C-section versus vaginal delivery after correction for gestational age at birth and time of scan, weight z-scores at birth, and other pertinent clinical variables. Our results elucidate neurobiological correlates of delivery method and explain the possible microstructural underpinnings of prior studies reporting adverse neurological outcomes in association with C-section delivery.

## METHODS

### Subject Selection

We reviewed the imaging and electronic health records of all infants who had brain MRI performed within 3 months of birth between January 2013 and March 2021 at our center. Data access and retrieval were performed on March 31, 2021. The authors had access to information that could identify individual participants during and after data collection. Preterm infants at our institution routinely undergo brain MRI at term equivalent age or prior to discharge. This predischarge MRI scan includes DTI series at the end, if tolerated by the infant. We included infants who (1) were born between 24- and 37-weeks gestational age at birth, (2) had no structural or signal abnormalities on conventional brain MRI series, (3) had DTI performed as part of their MRI study, and (4) had sufficient information in the health record regarding: delivery methods, indication of early delivery, gestational age at birth and time of scan, birth weight, and 1- and 5-minute APGAR scores. All birth weights were converted to Z-scores using the 2013 Fenton growth chart for preterm infants.(26) In addition, we excluded infants whose mothers engaged in documented substance use during pregnancy and those who were the product of a multiple gestations. We recorded the incidence of maternal preeclampsia, chorioamnionitis, premature rupture of membranes, need for infant intubation, need for infant therapeutic hypothermia, and indications for premature delivery for our included subjects.

As prior studies have identified low birth weight as a risk factor for delayed neurodevelopment and adverse neurological outcomes, a sub-cohort excluding infants born small for gestational age (SGA, birthweight z-score <−1.28) was identified to conduct additional analyses.(27, 28) All experiments and methods were performed in accordance with relevant guidelines and regulations. The study protocol was approved by the Yale University Human Research Protection Program Institutional Review Boards and the need for consent was waived due to the retrospective nature of the study.

### Image acquisition and pre-processing

MRI scans were performed on a Siemens 3 T Skyra scanner. DTI series were obtained using a single-shot echoplanar image sequence with the following parameters: repeat time = 10,200 ms, echo time = 94ms, flip angle = 90 degrees, field of view = 18x18 cm, slice thickness = 2.5mm, matrix = 128x128, including a single b = 0 and 30 noncolinear direction b = 1000 s/mm^2^ acquisitions. Motion artifact for neonatal MRI is reduced at our institution preferentially using the “feed-and-swaddle” technique with anesthetic sedation being used as a second line option.(29) Preprocessing of DTI scans including corrections for susceptibility distortions, eddy currents, and subject movement as well as generation of fractional anisotropy (FA), mean diffusivity (MD), radial diffusivity (RD), and axial diffusivity (AD) maps were performed using FMRIB’s Diffusion Toolbox (FDT) in FMRIB’s Software Library (FSL). All DTI metric maps were visually inspected for quality control prior to further analysis.

### Tract based spatial statistics (TBSS) and voxel-wise general linear model (GLM) analysis

The TBSS toolbox in FSL was used for voxel-wise statistical analysis of DTI metrics along WM tracts. As a part of this process, the most representative FA map of the cohorts was first identified by linearly co-registering each FA map to all other FA maps. This representative FA map was then co-registered to the MNI-152 standard brain space. The FA maps of all other subjects were co-registered to MNI-152 by combining the co-registration to the representative FA map and the co-registration of the representative map to MNI-152. This process was repeated to bring MD, RD, and AD maps to the MNI-152 space. A skeletonized WM tract map across all subjects was generated by averaging the aligned FA maps using a threshold value of 0.1. Then, we used the “randomise” tool in FSL for GLM analyses to examine the association of delivery method with DTI metric values across WM tracts while controlling for gestational age at birth, gestational age at scan, 5-minute APGAR, birth weight Z-score, presence of preeclampsia, and presence of chorioamnionitis as covariates. The 5-minute APGAR was chosen due to more prevalent availability in our dataset compared to 10-minute APGAR and its established association with outcomes.(30) As birth weight Z-score and presence of preeclampsia differed significantly between infants delivered vaginally and via C-section (Table 1), we performed additional analyses to examine the association of birth weight Z-score and presence of preeclampsia with DTI metrics after controlling for delivery method, gestational age at birth, gestational age at scan, 5-minute APGAR, and presence of chorioamnionitis as covariates. Additionally, as prior reports have described the association between general anesthesia use during C-section delivery and infant neurodevelopment, particularly with the use of inhaled anesthetics, we also analyzed the association between general anesthesia use during delivery and DTI metrics after controlling for delivery method, gestational age at birth, gestational age at scan, 5-minute APGAR, birth weight Z-score, presence of preeclampsia, and presence of chorioamnionitis as covariates.(31, 32) We applied 5000 permutations and threshold-free cluster enhancements while correcting for multiple comparisons in voxel-wise analysis. Results of the GLM analyses were visualized by overlaying a color-coded map of p-values (windowed for p-values < 0.05) onto the MNI-152 brain space template. Similar TBSS and GLM analyses were repeated for the sub-cohort excluding infants born small for gestational age.

### Tract-specific linear regression analysis

The results of our GLM analyses were further verified by conducting additional linear regression analyses across 48 WM tracts based on the John’s Hopkins University (JHU) atlas.(23) For every subject, the means of non-zero DTI metric values were calculated in each of the 48 WM tracts from JHU atlas using FSL. Then, we applied linear regression analyses to determine the association of delivery method with mean DTI metric values in each WM tract while controlling for gestational age at birth, gestational age at scan, 5-minute APGAR, birth weight Z-score, presence of preeclampsia, and presence of chorioamnionitis as covariates. Similar tract-specific linear regression analyses were repeated for the sub-cohort excluding infants born small for gestational age.

### Statistics

The data are presented as mean ± standard deviation, median (interquartile range) or frequency (percentage) as appropriate. The “stats” package in R was used to determine if significant differences existed with regards to clinical variables between infants delivered vaginally and via C-section. A two-sample t-test was used for continuous variables, a Wilcoxon rank-sum test was used for APGAR scores, and a chi-squared test was used for binary variables. R was also used to conduct tract-specific linear regression analyses.

## RESULTS

### Subject characteristics

**Figure 1:**
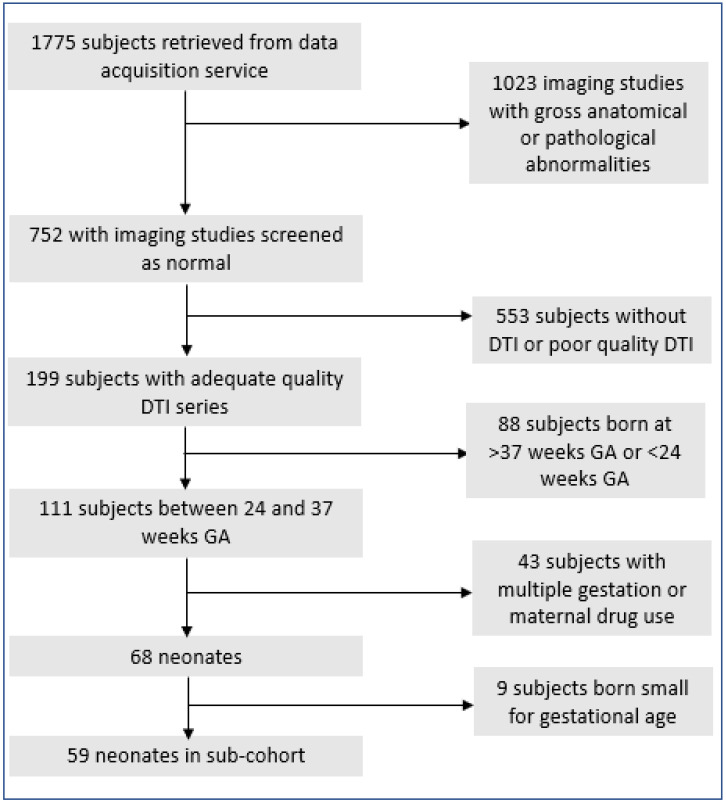
Inclusion flowchart. DTI = Diffusion tensor imaging. GA = gestational age.

Figure 1 depicts the flow chart for the inclusion of 68 preterm infants for this study as well as the 59 infants included in sub-cohort analyses. Our primary cohort consisted of 23 infants delivered vaginally and 45 infants delivered via C-section. Table 1 summarizes the clinical demographics of these infants. Of note, birth weight Z-score and presence of preeclampsia differed significantly between infants delivered vaginally and via C-section. The most common reason for preterm delivery in infants delivered vaginally was preterm labor (n = 18). Other reasons for preterm vaginal delivery included preterm labor with premature rupture of membranes (n = 4) or induction for chorioamnionitis (n = 1). The most common indication for preterm delivery via C-section were breech presentation (n = 13). Among those requiring C-section delivery due to breech presentation, preterm delivery was needed due to premature rupture of membranes (n = 6), preterm labor (n = 5), Hemolysis, Elevated Liver enzymes, and Low Platelets (HELLP) syndrome (n = 1), and failed external version (n = 1). Other indications for preterm C-section delivery included fetal heart rate abnormalities (n = 9), severe or worsening preeclampsia or HELLP syndrome (n = 11), placenta previa or accreta (n = 4), repeat C-section (n = 3), cord prolapse (n = 2), elective C-section (n = 1), arrested growth and absent or reversed umbilical artery doppler flow (n = 1), or preterm labor with history of prior uterine surgery (n = 1). In 13 of the 45 C-section deliveries, general anesthesia was used. For all infants whose maternal charts contained detailed anesthesia information (n = 11), sevoflurane was used as the inhaled anesthetic of choice and in 4 deliveries nitrous oxide was used as an additional inhaled anesthetic.

### Relationship of preterm infants’ delivery method with WM microstructure

**Figure 2:**
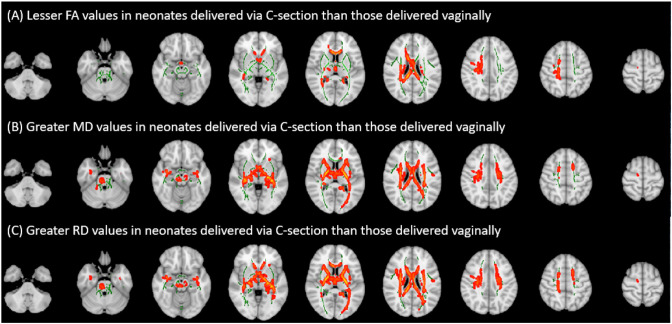
Voxel-wise general linear model analyses of the association of delivery method with (A) fractional anisotropy (FA), (B) mean diffusivity (MD), (C) and radial diffusivity (RD) values after controlling for gestational age at birth, gestational age at scan, 5-minute APGAR, birth weight Z-score, presence of preeclampsia, and presence of chorioamnionitis as covariates. Green areas depict white matter FA skeletons derived from tract based spatial statistics; red areas depict regions where delivery method was significantly (p<0.05) associated with corresponding diffusion tensor metrics.

Figure 2 depicts the results of the voxel-wise GLM analyses for the association of delivery method with FA, MD, and RD values after controlling for gestational age at birth, gestational age at scan, 5-minute APGAR, birth weight Z-score, presence of preeclampsia, and presence of chorioamnionitis as covariates. These results were further confirmed by tract-specific analyses (Tables 2-4). FA values in the corpus callosum, left internal capsule, right coronal radiata, and right tapetum were found to be significantly lower in infants delivered via C-section compared to those delivered vaginally (Fig. 2A and Table 2). MD values in the corpus callosum, cerebellar peduncle, left retrolenticular internal capsule, superior corona radiata, and fornix were found to be significantly higher in infants delivered via C-section than those delivered vaginally (Fig. 2B and Table 3). RD values in the corpus callosum, cerebellar peduncle, internal capsule, superior corona radiata, left cingulum, fornix, and left superior fronto-occipital fasciculus were significantly higher in infants delivered via C-section than those delivered vaginally (Fig. 2C and Table 4). There was no significant association of delivery method with AD values on voxel-wise or tract-specific analyses.

#### Relationship of preterm infants’ birth weight, maternal preeclampsia, and general anesthesia use during delivery with WM microstructure

Despite differing significantly between the two experimental groups, neither birth weight Z-score nor presence of maternal preeclampsia were significantly associated with any DTI metrics on voxel-wise GLM analysis or tract-specific regression after adjusting for delivery mode and other covariates listed above. Use of general anesthesia during delivery was also not significantly associated with any DTI metrics on voxel-wise GLM analysis or tract-specific regression after adjusting for delivery mode and additional covariates.

### Relationship of delivery method with WM microstructure after excluding small-for-gestational-age preterm infants

Nine infants born SGA (8 delivered by C-section and 1 delivered vaginally) were excluded from the sub-cohort. Supplemental Table 1 summarizes the clinical demographics of the infants included in the sub-cohort. Of note, birth weight Z-score differed significantly between infants delivered vaginally and via C-section in the sub-cohort. The excluded infant delivered vaginally required preterm delivery due to preterm labor. Indications for preterm C-section delivery among the excluded infants included severe or worsening preeclampsia or HELLP syndrome (n = 5), fetal heart rate abnormalities (n = 2), and breech presentation with failed external version (n = 1). Of the excluded C-section deliveries, one required the use of general anesthesia.

Supplemental Fig. 1 depicts the results of the voxel-wise GLM analyses for the association of delivery method with FA and RD values in the sub-cohort after controlling for gestational age at birth, gestational age at scan, 5-minute APGAR, birth weight Z-score, presence of preeclampsia, and presence of chorioamnionitis as covariates. These results were further confirmed by tract-specific analyses (Supplemental Tables 2 and 4). FA values in the body of the corpus callosum, corona radiata, and right tapetum were found to be significantly lower in infants delivered via C-section compared to those delivered vaginally (Supplemental Fig. 1A and Supplemental Table 2). RD values in the pontine crossing tract, corpus callosum, cerebellar peduncle, right posterior corona radiata, fornix, and stria terminalis were significantly higher in infants delivered via C-section than those delivered vaginally (Supplemental Fig. 1B and Supplemental Table 4). GLM analysis did not reveal any significant association between MD values and delivery method but MD values in pontine crossing tract, cerebellar peduncle, left retrolenticular internal capsule, right posterior corona radiata, fornix, stria terminalis, and right superior longitudinal fasciculus were found to be significantly higher in infants delivered via C-section than those delivered vaginally on tract-specific analysis (Supplemental Table 3). There was no significant association of delivery method with AD values on voxel-wise or tract-specific analyses. Birth weight z-score, maternal preeclampsia, and use of general anesthesia during delivery were not significantly associated with any DTI metrics in the sub-cohort after adjusting for delivery method and additional covariates.

## DISCUSSION

In both voxel-wise GLM and tract-based linear regression analyses, preterm infants requiring C-section delivery had reduced WM microstructural integrity compared to those delivered vaginally, most prominently in the corpus callosum, internal capsule, and corona radiata. Notably, the difference between delivery mode of preterm infants was found after adjusting for gestational age at birth, gestational age at scan, 5-minute APGAR, birth weight Z-score, presence of preeclampsia, and presence of chorioamnionitis as covariates – and after excluding infants from multiple gestations and those with any maternal history of drug use. Although birthweight Z-score and presence of maternal preeclampsia differed significantly between infants delivered vaginally and via C-section, additional voxel-wise GLM analyses found no significant association between these risk factors and DTI metrics after controlling for delivery method and other clinical variables. In the sub-cohort excluding SGA infants, areas of significant association between delivery method and DTI metrics were limited compared to the primary cohort but still included pertinent WM tracts such as the corpus callosum, internal capsule, and corona radiata.

Our results provide the neurobiological indications and early WM microstructural correlates for clinical studies reporting that preterm infants who require C-section are at risk for adverse neurodevelopmental outcome. The difference in early WM microstructural development may be a consequence of multiple factors necessitating C-section delivery; nevertheless, our findings highlight the higher risks of neurodevelopmental delay among preterm infants delivered via C-section and the need for close follow-up and potential intervention to mitigate these risks in this vulnerable cohort.

The myelination of WM tracts as infants age reduces the random diffusion of water molecules and results in alignment of diffusion along WM tracts. Thus, changes in DTI metrics such as increased FA and reduced MD and RD are corollaries for WM maturation.(33) DTI metrics have also been shown to predict neurological outcome, with studies reporting associations between DTI metrics measured during term-equivalent scans and cognitive, motor, and visual outcomes in childhood and adolescence.(34, 35) Of note, some have reported associations between the development of cerebral palsy and other motor abnormalities and term equivalent DTI metrics in the same WM regions where we identified significant association between delivery method and DTI metrics such as the corpus callosum and internal capsule. (36, 37) Thus, term-equivalent DTI can potentially help identify infants at risk for adverse neurological outcomes with regards to their method of delivery, especially when neurological exam and standard neuroimaging might otherwise be unremarkable.

The relationship between delivery method and infant brain development has not been extensively studied. Deoni et. al have compared the DTI metrics between term infants delivered via C-section (n = 11) versus vaginally (n = 32).(25) Similar to our results, they reported higher FA values in WM tracts of term infants delivered vaginally compared to those delivered via C-section. We found higher MD and RD in addition to reduced FA in preterm infants delivered via C-section. Deoni et. al also noted slight variations in results with the inclusion of additional covariates, suggesting that factors beyond delivery method could be contributing to perceived differences in WM maturation. We attempted to address this by controlling for clinical variables known to be independently associated with WM maturation in infants (gestational age) in addition to those we found differed significantly between our two experimental groups (birthweight z-score and maternal preeclampsia), among others.(23) As prior reports have raised concern that exposure to general anesthesia, particularly inhaled agents such as sevoflurane, may contribute to delayed neurodevelopment and adverse long-term neurological outcomes, we also confirmed that exposure to general anesthesia during delivery was not independently associated with WM maturation in our cohort. (31, 32, 38, 39) Of note, Deoni et. al found no difference in WM tract DTI metrics when comparing separate cohorts of eight-year-old children delivered via C-section (n = 23) versus vaginally (n = 37). Thus, they proposed that WM microstructural differences in association with delivery mode may only be present in early (pre-discharge) MRIs and not resolve at older age – although they analyzed different children’s cohorts from infants.

As we demonstrate the relationship between delivery method and WM maturation on neuroimaging, the question of how delivery method may influence early brain development arises. It has been suggested that vaginal delivery is an important contributor to the normal development of infants’ gut microbiome and that alterations in this process (due to C-section) can disturb normal brain development.(40, 41) The impact of differential infant hormonal expression between vaginal and C-section deliveries on early brain development has also been proposed.(42) However, these explanations are complicated by studies reporting contrasting results regarding the association of delivery method with neurodevelopmental disorders.(19, 43, 44) Even among the numerous studies suggesting that delivery method may be associated with neurological outcomes, differences in methodology (such as additional clinical covariates that were controlled) and characteristics of experimental cohorts among prior reports complicate interpretation. Additionally, some investigators report that delivery method may be associated with neurological outcome only in the context of additional factors such as infant gender, APGAR scores, multiple gestations, and use of general anesthesia during C-section.(12, 16, 17, 32) Given the many maternal and infant factors that may influence need for C-section versus vaginal delivery, it is challenging to control for all covariates that may influence brain development independently or cooperatively with delivery method. Nevertheless, our study provides WM microstructural correlates for prior clinical studies reporting that preterm infants requiring C-section are at higher risk of neurodevelopmental delay. While our findings may be related to multifactorial circumstances necessitating C-section delivery, they highlight the need for close neurological follow up and timely interventions in these vulnerable children.

Exclusion of infants born SGA in the sub-cohort analyses limited the regions of apparent significant association between delivery method and DTI metrics when compared to primary cohort analyses. This suggests that the results of our primary analyses could be partially mediated by the lower birth weight and birth weight z-score among neonates born by C-section versus vaginally in the primary cohort. Indeed, prior reports have described the association between birth weight and DTI metrics as well as long-term neurological outcomes.(45-47) However, birth weight does not entirely explain the results of our primary analyses as evidenced by lack of significant independent association between birth-weight z-score and DTI metrics in primary and sub-cohort analyses as well as sustained significant associations between delivery method and DTI metrics in the corpus callosum, internal capsule, and corona radiata in our sub-cohort analyses.

Our study is limited by its retrospective, single center design and small sample size. Our small sample size also limits the power of our study in including multiple covariates, which may complicate the interpretations of our analyses on the independent associations of birthweight z-score, maternal preeclampsia, and general anesthesia use during delivery with DTI metrics. The small sample size especially limited the sub-cohort analysis after exclusion of 9 SGA preterm infants. DTI metric analysis can be influenced by acquisition protocol and timing, as well as processing methods used to generate DTI metric maps. Although we controlled for several demographic and clinical variables, including those that differed significantly between experimental groups, we could not account for all possible covariates that may influence delivery method and explain observed differences in DTI metrics between the experimental groups in this study. Of note, recent work has suggested that intrauterine inflammation impacts fetal and infant neurodevelopment and although we controlled for the presence of chorioamnionitis as a covariate, we did not collect placental pathology data to assess for other causes of intrauterine inflammation.(48, 49) We also described various indications for respective delivery method but could not control for all such indications given the widely varying delivery indications in our cohort. Finally, long-term neurological outcomes were not available in our dataset to corroborate clinical correlates of DTI findings.

## CONCLUSION

Preterm infants requiring C-section section delivery, compared to those delivered vaginally, have neuroimaging markers of delayed WM maturation on predischarge MRI scans, most prominently in the corpus callosum, internal capsule, and corona radiata independent of gestational age at birth, gestational age at scan, 5-minute APGAR, birth weight Z-score, maternal preeclampsia, and chorioamnionitis. Possible confounders of birth weight z-score, rates of maternal preeclampsia, and use of general anesthesia during C-section delivery are not independently associated with WM maturation in this cohort. Lastly, our primary results may be partially, but not entirely, mediated by lower birth weights among infants delivered by C-section versus vaginally. These findings suggest that preterm infants requiring C-section are at risk for delays in WM maturation and thus may require close neurologic follow up to identify and mitigate adverse long-term neurological outcomes.

## Figures and Tables

**Table 1:** Demographic characteristics of infants included in the study (n = 68, 23 vaginal delivery and 45 C-section)

Clinical variable	Vaginal delivery	C-section	p-value
Gestational age at birth (weeks)	28.45 ± 3.66	29.29 ± 3.91	0.38
Gestational age at scan (weeks)	38.13 ± 2.53	37.77 ± 2.75	0.59
Birth Weight (grams)	1305.39 ± 564.31	1284.67 ± 726.63	0.9
Birth Weight Z-score	0.56 ± 0.81	0.263 ± 1.00	**0.0006**
Maternal Age (years)	29.05 ± 6.37	31.25 ± 5.98	0.20
1-minute APGAR	7 (4.5-8)	5 (4–8)	0.11
5-minute APGAR	8 (7–9)	8 (6–9)	0.82
Blood glucose	71.78 ± 26.81	70.59 ± 40.75	0.89
Hematocrit (%)	43.36 ± 5.09	44.64 ± 7.70	0.44
Sex (males)	65.22%	55.56%	0.61
Preeclampsia	0.00%	33.33%	**0.005**
Intubated	43.48%	55.56%	0.49
Chorioamnionitis	4.35%	6.67%	1.00
Premature rupture of membranes	17.39%	26.67%	0.58
Received Therapeutic Hypothermia	0.00%	2.22%	1.00

Note: Data are reported as mean ± standard deviation, median (interquartile range), or frequency as a percent. p-values represent the outcome of an appropriate statistical test comparing the clinical variables between infants born vaginally and via C-section.

**Table 2:** Tract-specific linear regression analysis of the association of delivery method with fractional anisotropy values after controlling for gestational age at birth, gestational age at scan, 5-minute APGAR, birth weight Z-score, presence of preeclampsia, and presence of chorioamnionitis as covariates.

Tract	Coefficient (95% Confidence Interval)	p-value
Middle cerebellar peduncle	−4.49E-03 (−1.51E-02 to 6.14E-03)	0.40
Pontine crossing tract	−3.80E-03 (−2.11E-02 to 1.35E-02)	0.66
Genu of corpus callosum	−1.32E-02 (−2.51E-02 to −1.39E-03)	**0.03**
Body of corpus callosum	−1.57E-02 (−2.74E-02 to −4.09E-03)	**0.01**
Splenium of corpus callosum	−8.92E-03 (−2.04E-02 to 2.58E-03)	0.13
Fornix	−1.18E-02 (−3.11E-02 to 7.46E-03)	0.22
Corticospinal tract R	1.10E-03 (−1.27E-02 to 1.49E-02)	0.87
Corticospinal tract L	−9.82E-03 (−2.22E-02 to 2.60E-03)	0.12
Medial lemniscus R	1.10E-02 (−1.24E-02 to 3.44E-02)	0.35
Medial lemniscus L	−1.72E-03 (−2.15E-02 to 1.81E-02)	0.86
Inferior cerebellar peduncle R	−5.43E-03 (−2.09E-02 to 1.01E-02)	0.49
Inferior cerebellar peduncle L	−8.11E-03 (−2.37E-02 to 7.52E-03)	0.30
Superior cerebellar peduncle R	−6.33E-03 (−1.89E-02 to 6.28E-03)	0.32
Superior cerebellar peduncle L	−4.13E-03 (−1.69E-02 to 8.66E-03)	0.52
Cerebral peduncle R	−1.65E-03 (−1.37E-02 to 1.04E-02)	0.79
Cerebral peduncle L	5.61E-04 (−1.22E-02 to 1.33E-02)	0.93
Anterior limb of internal capsule R	−4.59E-03 (−1.47E-02 to 5.49E-03)	0.37
Anterior limb of internal capsule L	−1.42E-02 (−2.71E-02 to −1.30E-03)	**0.03**
Posterior limb of internal capsule R	−1.27E-02 (−2.55E-02 to 2.12E-04)	0.05
Posterior limb of internal capsule L	−1.38E-02 (−2.75E-02 to −9.99E-06)	**0.0498**
Retrolenticular part of internal capsule R	−7.88E-03 (−2.20E-02 to 6.20E-03)	0.27
Retrolenticular part of internal capsule L	−7.00E-03 (−2.20E-02 to 8.01E-03)	0.35
Anterior corona radiata R	−8.39E-03 (−1.89E-02 to 2.12E-03)	0.12
Anterior corona radiata L	−4.71E-03 (−1.47E-02 to 5.23E-03)	0.35
Superior corona radiata R	−1.59E-02 (−2.70E-02 to −4.71E-03)	**0.01**
Superior corona radiata L	−1.07E-02 (−2.28E-02 to 1.52E-03)	0.09
Posterior corona radiata R	−1.20E-02 (−2.26E-02 to −1.53E-03)	**0.03**
Posterior corona radiata L	−9.83E-03 (−2.26E-02 to 2.90E-03)	0.13
Posterior thalamic radiation R	−4.49E-03 (−1.42E-02 to 5.25E-03)	0.36
Posterior thalamic radiation L	−8.64E-03 (−2.16E-02 to 4.34E-03)	0.19
Sagittal stratum R	−8.06E-03 (−2.05E-02 to 4.39E-03)	0.20
Sagittal stratum L	−8.99E-03 (−2.10E-02 to 3.03E-03)	0.14
External capsule R	−4.08E-03 (−1.33E-02 to 5.12E-03)	0.38
External capsule L	−2.90E-03 (−1.45E-02 to 8.67E-03)	0.62
Cingulum (cingulate gyrus) R	−2.14E-03 (−1.00E-02 to 5.72E-03)	0.59
Cingulum (cingulate gyrus) L	−6.43E-03 (−1.39E-02 to 1.02E-03)	0.09
Cingulum (hippocampus) R	−5.03E-03 (−1.65E-02 to 6.39E-03)	0.38
Cingulum (hippocampus) L	5.27E-03 (−7.48E-03 to 1.80E-02)	0.41
Fornix (cres) / Stria terminalis R	−8.04E-03 (−1.94E-02 to 3.32E-03)	0.16
Fornix (cres) / Stria terminalis L	−7.78E-03 (−1.95E-02 to 3.96E-03)	0.19
Superior longitudinal fasciculus R	−3.49E-03 (−1.25E-02 to 5.48E-03)	0.44
Superior longitudinal fasciculus L	−6.20E-03 (−1.60E-02 to 3.61E-03)	0.21
Superior fronto-occipital fasciculus R	−4.32E-03 (−1.53E-02 to 6.65E-03)	0.43
Superior fronto-occipital fasciculus L	−6.16E-03 (−2.04E-02 to 8.12E-03)	0.39
Uncinate fasciculus R	1.91E-04 (−1.45E-02 to 1.49E-02)	0.98
Uncinate fasciculus L	−8.53E-03 (−2.21E-02 to 5.01E-03)	0.21
Tapetum R	−1.89E-02 (−3.27E-02 to −5.21E-03)	**0.01**
Tapetum L	−8.33E-03 (−2.69E-02 to 1.02E-02)	0.37

Note- Coefficients of regression, upper and lower bounds of a 95% confidence interval, and p-values are provided for regressions performed in each WM tract. Significant (< 0.05) p-values are in bold.

**Table 3:** Tract-specific linear regression analysis of the association of delivery method with mean diffusivity values after controlling for gestational age at birth, gestational age at scan, 5-minute APGAR, birth weight Z-score, presence of preeclampsia, and presence of chorioamnionitis as covariates.

Tract	Coefficient (95% Confidence Interval)	p-value
Middle cerebellar peduncle	2.52E-05 (−5.28E-05 to 1.03E-04)	0.52
Pontine crossing tract	5.40E-05 (−1.27E-05 to 1.21E-04)	0.11
Genu of corpus callosum	2.64E-05 (−5.25E-06 to 5.81E-05)	0.10
Body of corpus callosum	5.28E-05 (1.65E-05 to 8.92E-05)	**0.01**
Splenium of corpus callosum	6.36E-05 (1.11E-05 to 1.16E-04)	**0.02**
Fornix	7.95E-05 (−6.41E-05 to 2.23E-04)	0.27
Corticospinal tract R	−3.41E-06 (−1.62E-04 to 1.55E-04)	0.97
Corticospinal tract L	4.16E-05 (−1.18E-04 to 2.02E-04)	0.60
Medial lemniscus R	8.20E-05 (−6.64E-05 to 2.30E-04)	0.27
Medial lemniscus L	3.87E-05 (−1.03E-04 to 1.81E-04)	0.59
Inferior cerebellar peduncle R	1.18E-04 (2.41E-05 to 2.12E-04)	**0.01**
Inferior cerebellar peduncle L	1.15E-04 (−6.65E-06 to 2.37E-04)	0.06
Superior cerebellar peduncle R	9.67E-05 (1.12E-05 to 1.82E-04)	**0.03**
Superior cerebellar peduncle L	1.01E-04 (1.74E-05 to 1.84E-04)	**0.02**
Cerebral peduncle R	−6.50E-06 (−6.05E-05 to 4.75E-05)	0.81
Cerebral peduncle L	2.31E-05 (−2.29E-05 to 6.91E-05)	0.32
Anterior limb of internal capsule R	2.62E-05 (−1.56E-05 to 6.80E-05)	0.21
Anterior limb of internal capsule L	4.62E-05 (−4.12E-06 to 9.64E-05)	0.07
Posterior limb of internal capsule R	2.20E-05 (−1.36E-06 to 4.53E-05)	0.06
Posterior limb of internal capsule L	2.23E-05 (−2.93E-06 to 4.76E-05)	0.08
Retrolenticular part of internal capsule R	2.58E-05 (−5.40E-06 to 5.71E-05)	0.10
Retrolenticular part of internal capsule L	3.53E-05 (5.84E-06 to 6.48E-05)	**0.02**
Anterior corona radiata R	4.21E-05 (−1.22E-05 to 9.64E-05)	0.13
Anterior corona radiata L	4.99E-05 (−7.21E-06 to 1.07E-04)	0.09
Superior corona radiata R	5.36E-05 (6.73E-06 to 1.01E-04)	**0.03**
Superior corona radiata L	6.01E-05 (9.70E-06 to 1.10E-04)	**0.02**
Posterior corona radiata R	4.30E-05 (−1.22E-05 to 9.82E-05)	0.12
Posterior corona radiata L	4.52E-05 (−1.96E-05 to 1.10E-04)	0.17
Posterior thalamic radiation R	2.32E-05 (−1.80E-05 to 6.44E-05)	0.26
Posterior thalamic radiation L	4.10E-05 (−2.20E-05 to 1.04E-04)	0.20
Sagittal stratum R	2.62E-05 (−1.74E-05 to 6.98E-05)	0.23
Sagittal stratum L	3.41E-05 (−1.29E-05 to 8.12E-05)	0.15
External capsule R	3.09E-05 (−5.56E-06 to 6.73E-05)	0.10
External capsule L	2.01E-05 (−1.40E-05 to 5.42E-05)	0.24
Cingulum (cingulate gyrus) R	2.17E-05 (−5.79E-06 to 4.91E-05)	0.12
Cingulum (cingulate gyrus) L	2.56E-05 (−3.23E-06 to 5.45E-05)	0.08
Cingulum (hippocampus) R	2.81E-05 (−5.04E-06 to 6.13E-05)	0.10
Cingulum (hippocampus) L	1.50E-05 (−2.53E-05 to 5.53E-05)	0.46
Fornix (cres) / Stria terminalis R	4.76E-05 (6.77E-06 to 8.85E-05)	**0.02**
Fornix (cres) / Stria terminalis L	7.27E-05 (2.39E-05 to 1.21E-04)	**0.004**
Superior longitudinal fasciculus R	3.54E-05 (−4.74E-06 to 7.54E-05)	0.08
Superior longitudinal fasciculus L	4.00E-05 (−7.92E-06 to 8.79E-05)	0.10
Superior fronto-occipital fasciculus R	3.96E-05 (−3.09E-05 to 1.10E-04)	0.27
Superior fronto-occipital fasciculus L	6.60E-05 (−2.42E-06 to 1.34E-04)	0.06
Uncinate fasciculus R	1.38E-05 (−6.52E-05 to 9.28E-05)	0.73
Uncinate fasciculus L	7.00E-05 (−1.87E-05 to 1.59E-04)	0.12
Tapetum R	2.69E-05 (−1.01E-04 to 1.55E-04)	0.67
Tapetum L	2.74E-05 (−1.87E-04 to 2.42E-04)	0.80

Note- Coefficients of regression, upper and lower bounds of a 95% confidence interval, and p-values are provided for regressions performed in each WM tract. Significant (< 0.05) p-values are in bold.

**Table 4:** Tract-specific linear regression analysis of the association of delivery method with radial diffusivity values after controlling for gestational age at birth, gestational age at scan, 5-minute APGAR, birth weight Z-score, presence of preeclampsia, and presence of chorioamnionitis as covariates.

Tract	Coefficient (95% Confidence Interval)	p-value
Middle cerebellar peduncle	2.60E-05 (−4.94E-05 to 1.01E-04)	0.49
Pontine crossing tract	5.33E-05 (−1.15E-05 to 1.18E-04)	0.11
Genu of corpus callosum	3.43E-05 (4.20E-07 to 6.81E-05)	**0.05**
Body of corpus callosum	6.18E-05 (2.32E-05 to 1.00E-04)	**0.002**
Splenium of corpus callosum	6.48E-05 (1.26E-05 to 1.17E-04)	**0.02**
Fornix	8.69E-05 (−5.42E-05 to 2.28E-04)	0.22
Corticospinal tract R	−7.12E-06 (−1.58E-04 to 1.44E-04)	0.93
Corticospinal tract L	4.50E-05 (−1.06E-04 to 1.96E-04)	0.55
Medial lemniscus R	6.94E-05 (−7.60E-05 to 2.15E-04)	0.34
Medial lemniscus L	3.65E-05 (−1.02E-04 to 1.75E-04)	0.60
Inferior cerebellar peduncle R	1.15E-04 (2.48E-05 to 2.05E-04)	**0.01**
Inferior cerebellar peduncle L	1.13E-04 (−9.01E-07 to 2.28E-04)	**0.05**
Superior cerebellar peduncle R	9.34E-05 (1.33E-05 to 1.74E-04)	**0.02**
Superior cerebellar peduncle L	9.63E-05 (1.83E-05 to 1.74E-04)	**0.02**
Cerebral peduncle R	−4.05E-06 (−5.57E-05 to 4.76E-05)	0.88
Cerebral peduncle L	1.99E-05 (−2.57E-05 to 6.54E-05)	0.39
Anterior limb of internal capsule R	2.71E-05 (−1.49E-05 to 6.92E-05)	0.20
Anterior limb of internal capsule L	5.09E-05 (2.65E-06 to 9.91E-05)	**0.04**
Posterior limb of internal capsule R	2.77E-05 (1.42E-06 to 5.40E-05)	**0.04**
Posterior limb of internal capsule L	2.82E-05 (5.62E-07 to 5.58E-05)	**0.046**
Retrolenticular part of internal capsule R	2.62E-05 (−8.15E-06 to 6.05E-05)	0.13
Retrolenticular part of internal capsule L	3.33E-05 (−4.94E-07 to 6.72E-05)	0.05
Anterior corona radiata R	4.62E-05 (−1.08E-05 to 1.03E-04)	0.11
Anterior corona radiata L	5.35E-05 (−7.47E-06 to 1.15E-04)	0.08
Superior corona radiata R	6.09E-05 (1.20E-05 to 1.10E-04)	**0.02**
Superior corona radiata L	6.22E-05 (9.97E-06 to 1.14E-04)	**0.02**
Posterior corona radiata R	4.87E-05 (−8.14E-06 to 1.06E-04)	0.09
Posterior corona radiata L	4.79E-05 (−1.90E-05 to 1.15E-04)	0.16
Posterior thalamic radiation R	2.37E-05 (−1.49E-05 to 6.23E-05)	0.22
Posterior thalamic radiation L	4.32E-05 (−1.67E-05 to 1.03E-04)	0.15
Sagittal stratum R	3.06E-05 (−1.63E-05 to 7.74E-05)	0.20
Sagittal stratum L	3.75E-05 (−1.19E-05 to 8.69E-05)	0.13
External capsule R	3.06E-05 (−4.81E-06 to 6.59E-05)	0.09
External capsule L	2.12E-05 (−1.48E-05 to 5.73E-05)	0.24
Cingulum (cingulate gyrus) R	2.14E-05 (−4.86E-06 to 4.77E-05)	0.11
Cingulum (cingulate gyrus) L	2.86E-05 (2.06E-06 to 5.51E-05)	**0.04**
Cingulum (hippocampus) R	2.96E-05 (−3.42E-06 to 6.26E-05)	0.08
Cingulum (hippocampus) L	1.05E-05 (−3.22E-05 to 5.31E-05)	0.63
Fornix (cres) / Stria terminalis R	4.79E-05 (7.80E-06 to 8.80E-05)	**0.02**
Fornix (cres) / Stria terminalis L	7.05E-05 (2.14E-05 to 1.20E-04)	**0.01**
Superior longitudinal fasciculus R	3.53E-05 (−5.49E-06 to 7.61E-05)	0.09
Superior longitudinal fasciculus L	4.08E-05 (−8.49E-06 to 9.01E-05)	0.10
Superior fronto-occipital fasciculus R	3.89E-05 (−2.60E-05 to 1.04E-04)	0.24
Superior fronto-occipital fasciculus L	6.49E-05 (2.47E-06 to 1.27E-04)	**0.04**
Uncinate fasciculus R	1.37E-05 (−6.65E-05 to 9.39E-05)	0.73
Uncinate fasciculus L	7.05E-05 (−1.74E-05 to 1.58E-04)	0.11
Tapetum R	4.29E-05 (−7.91E-05 to 1.65E-04)	0.48
Tapetum L	3.62E-05 (−1.79E-04 to 2.52E-04)	0.74

Note- Coefficients of regression, upper and lower bounds of a 95% confidence interval, and p-values are provided for regressions performed in each WM tract. Significant (< 0.05) p-values are in bold.

## Data Availability

The datasets generated during and/or analysed during the current study are available from the corresponding author on reasonable request.
